# Pentaspline Pulsed Field Ablation Versus High‐Power Short‐Duration/Very High‐Power Short‐Duration Radiofrequency Ablation in Atrial Fibrillation: A Meta‐Analysis

**DOI:** 10.1111/jce.16776

**Published:** 2025-07-09

**Authors:** Marco Valerio Mariani, Andrea Matteucci, Nicola Pierucci, Marta Palombi, Paolo Compagnucci, Raffaele Bruti, Pietro Cipollone, Sara Vinciullo, Sara Trivigno, Agostino Piro, Vincenzo Mirco La Fazia, Wael Saade, Cristina Chimenti, Giovanni Battista Forleo, Antonio Dello Russo, Claudio Pandozi, Andrea Natale, Carmine Dario Vizza, Carlo Lavalle

**Affiliations:** ^1^ Department of Cardiovascular, Respiratory, Nephrological Aenesthesiological and Geriatric Sciences “Sapienza” University of Rome Rome Italy; ^2^ Clinical and Rehabilitation Cardiology Division San Filippo Neri Hospital Rome Italy; ^3^ Cardiology and Arrhythmology Clinic University Hospital “Azienda Ospedaliero‐Universitaria delle Marche” Ancona Italy; ^4^ Depatrment of Biomedicine and Prevention, Division of Cardiology University of Tor Vergata Rome Italy; ^5^ Department of Internal Clinical Aenesthesiological and Cardiovascular Sciences “Sapienza” University of Rome Rome Italy; ^6^ Cardiology Unit Luigi Sacco University Hospital Milan Italy

**Keywords:** atrial fibrillation, high‐power short‐duration ablation, pulmonary vein isolation, pulsed field ablation, radiofrequency ablation

## Abstract

**Background:**

Pulsed field energy has been proposed as alternative to radiofrequency energy in atrial fibrillation (AF) ablation.

**Objective:**

To compare data from studies assessing AF ablation with pulsed field ablation (PFA) versus high‐power short‐duration (HPSD) or very HPSD (vHPSD) radiofrequency ablation (RFA) in terms of AF recurrence, procedure‐related complications, fluoroscopy and procedure times.

**Methods:**

A search of online scientific libraries (from inception to October 1, 2024) was performed. Six studies were considered eligible for the meta‐analysis totaling 1190 patients of whom 568 receiving PFA and 622 receiving HPHD/vHPSD RFA.

**Results:**

In patients with paroxysmal AF (PAF), a nonsignificant reduction of AF recurrence was related to PFA as compared to HPSD/vHPSD RFA (OR 0.74 [0.50; 1.11], *p* = 0.14, I^2^ 10%). In patients with persistent AF (PeAF) a nonsignificant reduction of AF recurrence was related to PFA as compared to HPSD/vHPSD RFA (OR of 0.68 [95%CI 0.35; 1.34], *p*‐value 0.27, I^2^ 10%). In the overall population PFA was associated with a significant reduction of AF recurrence at follow‐up (OR 0.65 [0.47; 0.90], *p* = 0.009, I^2^ 11%). No statistical differences were found among the groups in terms of total complications (OR 0.92, [0.45; 1.86], *p* = 0.81, I^2^ = 27%), stroke (*p* = 0.78), and cardiac tamponade (*p* = 0.80). PFA was associated with significantly longer fluoroscopy time (WMD 8.69 [5.64; 11.75], *p* < 0.001, I^2^ = 95%), but shorter procedure time (WMD −35.16 [ − 46.03; −24.28], *p* < 0.001, I^2^ = 89%) compared to HPSD/vHPSD RFA.

**Conclusion:**

PFA in AF patients is associated with similar efficacy and safety profiles as compared to HPSD/vHPSD RFA.

## Graphical abstract

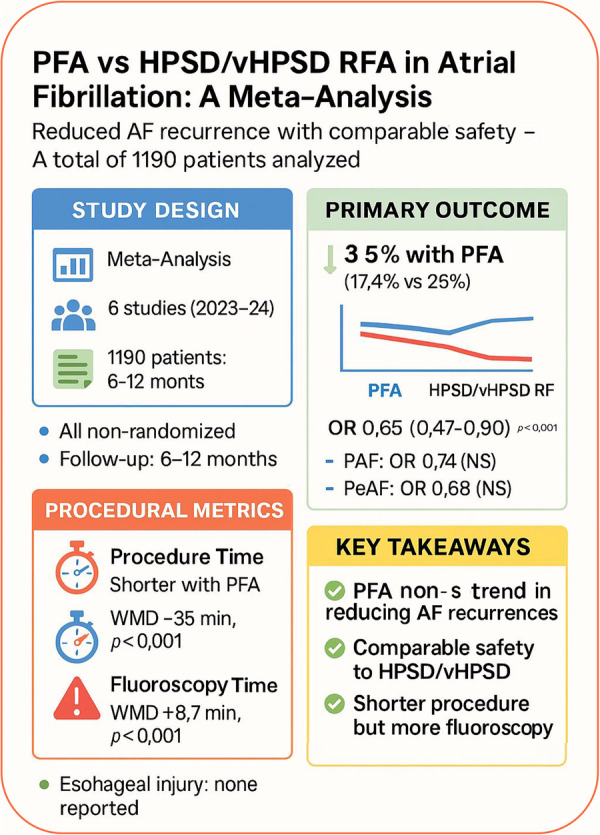

## 1 Introduction

Atrial fibrillation (AF) is the most commonly diagnosed arrhythmia in clinical practice [1], and is associated with increased risk of heart failure (HF) and thromboembolic events [2]. Rhythm control strategies proved to be more effective than rate control strategies in reducing AF burden and improving quality of life (QoL), as well as in decreasing the risk of adverse cardiovascular outcomes [3, 4]. In this context, AF catheter ablation was shown to be more effective than antiarrhythmic drugs (AADs) in maintaining sinus rhythm and improving symptoms, either as first‐line therapy or after AADs failure or intolerance [5, 6, 7, 8, 9].

Point‐by‐point pulmonary vein isolation (PVI) using conventional low‐power (25–40 W) radiofrequency ablation (RFA) has been the mainstay of AF ablation procedure. Nevertheless, RFA portends the risk of complications associated with the damage of collateral structures, as well is time‐consuming and requires adequate catheter stability and an optimal contact force (CF) over time to achieve durable, transmural lesions [10, 11]. Point‐by‐point based PVI was implemented by technological improvement in contact force sensing and ablation index (AI)‐guided RFA, as well as by the introduction of high‐power short‐duration (HPSD) and very HPSD (vHPSD) ablation protocol (with a maximum power of 90 W). These advancements have resulted in shorter procedure durations and alleged higher efficacy and safety as compared with low‐power long‐duration (LPLD) RFA [12]. In the last years, pulsed field ablation (PFA) has emerged as a groundbreaking tool for achieving PVI, relying on a non‐thermal energy source leading to cardiac myocyte electroporation [13, 14]. In recent studies and meta‐analyses, PFA has been associated with shorter procedure and left atrium (LA) dwell times, reduced risk of extracardiac complications and PV stenosis, and similar or superior efficacy outcomes as compared with LPLD RFA [15, 16, 17]. However, few studies directly compared PFA and HPSD/vHPSD RFA in AF ablation [18, 19, 20, 21, 22, 23], and whether there are differences in terms of safety, efficacy, and procedural metrics among the two ablation strategies is not established yet.

To clarify this issue, we performed a meta‐analysis of currently available studies comparing AF catheter ablation with PFA versus HPSD/vHPSD RFA in terms of AF recurrence, procedure‐related complications, fluoroscopy and procedure times.

## 2 Methods

### 2.1 Search Strategy, Selection Criteria and Outcomes

The present meta‐analysis was performed in accordance with the Preferred Reporting Items for Systematic Reviews and Meta‐analysis (PRISMA) statement [24].

An online search of Pubmed, Web of Science, Cochrane Registry, Scopus, and EMBASE libraries (from inception to October 1, 2024) was performed, in addition to manual screening. We used the following keywords: [atrial fibrillation]; [catheter ablation]; [(pulmonary vein isolation) OR (pulmonary vein ablation)]; [(radiofrequency ablation) OR (RFA)]; [(high‐power short‐duration ablation) OR (HPSD ablation)]; [(very high‐power short‐duration ablation) OR (vHPSD ablation)]; [(pulsed field ablation) OR (PFA) OR (electroporation)] in various combinations. No language restriction was applied. We included studies comparing PFA versus HSPD/vHPSD RFA in the treatment of AF. Reviews, editorials, letters, meta‐analysis, case reports and abstracts were excluded.

The following outcomes were evaluated: AF recurrence in overall population and in the subgroup of patients with PAF and persistent AF (PeAF), fluoroscopy and procedure times, procedure‐related complications (i.e. vascular access complications, thromboembolic events, cerebrovascular accidents, pulmonary vein stenosis, phrenic nerve injury, cardiac tamponade, coronary spasm, hemolysis, pericarditis). AF recurrence was defined as any episode of AF longer than 30 s after the immediate post ablation period (i.e. blanking period), regardless of symptoms.

Two independent reviewers (MVM and MP) screened all abstracts and titles to identify potentially eligible studies, of which full text was subsequently interrogated. Agreement of the two reviewers was required for eligibility of studies for analysis. Disagreements regarding the inclusion or the classification of a study were solved by a third reviewer (AM).

### 2.2 Data Extraction and Quality Assessment

Data extraction was performed by two reviewers (MVM and MP). For each study the following data were collected: first author and year of publication, study design, population size, AF pattern (PAF or PeAF), outcomes of interest (PAF recurrence, total AF recurrence, fluoroscopic time, procedural time, procedure‐related complications), antiarrhythmic therapy, ablative energy (PFA, HPSD/VHPSD RFA), ablative strategy (application duration and energy, PVI only ablation or PVI plus additional lesions), months of follow‐up, AF recurrence monitoring, patients' demographics (mean age, gender, AF pattern, mean left ventricular ejection fraction [LVEF]).

Study quality was evaluated by two reviewers (MVM and MP) using the Newcastle–Ottawa scale (NOS) for non‐randomized cohort studies [25]. Three categories were included in the analysis, with some of them having subcategories for assessment. Studies were subsequently classified into one of three categories: (i) high quality: 6–9 points; (ii) satisfactory quality: 3–5 points; and (iii) unsatisfactory quality: 0–2 points [25].

### 2.3 Statistical Analysis

Descriptive analysis was based on counts (percentages) for dichotomous and categorical variables and as mean ± standard deviation (SD) for continuous variables or number of cases (n). Statistical analysis was performed using Review Manager (RevMan version 5.3, the Cochrane Collaboration, 2014; Oxford, United Kingdom). Statistical heterogeneity on each outcome of interest was quantified using I^2^ statistic and the Cochrane Q test. Values of I^2^ statistic, ≤ 25%, 50%, and ≥ 75% indicated low, moderate, and high heterogeneity, respectively, whereas for Q statistic, substantial heterogeneity was defined as *p* < 0.1. Data were pooled using a random effect model, and the effect estimates chosen were the odd ratios (ORs) and weighted mean difference (WMD) with their corresponding 95% confidence interval (CIs), as needed. A sensitivity analysis was conducted using a fixed effect model and including studies with or without ablation beyond PVI. Moreover, a leave‐one‐out sensitivity analysis for the outcome AF recurrence at follow‐up was performed to show the independence of the results on one study alone. In addition, we evaluated the outcome AF recurrence dividing the studies based on follow‐up duration and on the basis of AAD use after the procedure. A *p*‐value < 0.05 was considered statistically significant.

## 3 Results

### 3.1 Study Selection and Patients' Characteristics

The literature search process identified 1009 studies (Supporting Information Figure A.1). After excluding duplicate publications, reviews, editorials, letters to editor, meta‐analysis, case reports and abstracts, 13 studies were fully reviewed, and 6 studies were considered eligible for the meta‐analysis [18, 19, 20, 21, 22, 23]; none of the selected studies were randomized controlled trials (RCTs). Baseline characteristics of the included studies and study population are summarized in Table 1. The overall population counted 1190 patients (568 receiving PFA and 622 receiving HPSD/vHPSD RFA); mean age was 66.4 ± 12.6 years, more than half of patients were male. The mean follow‐up went from 6 to 12 months, with 2 studies reporting a follow‐up of 6 months [21, 23], 1 study with a mean follow‐up of 7 months [18] and the last 3 studies with a mean follow‐up of 12 months [19, 20, 22]; the blanking period varied from 1 to 3 months, Badertscher et al. [18] did not report blanking period duration. Two studies did not report AADs use after index ablation [18, 21], whereas in the study by Dello Russo et al. [19], AADs were administered during the study period in less than 30% of patients. In the remaining studies AADs were discontinued immediately or 3 months after the procedure. Popa et al. [23] and Reinsch et al. [20] only included patients with PAF, while the last studies enrolled different rates of PAF patients, ranging from 30% to 67%. Overall, 905 PAF patients and 285 patients with persistent AF (PersAF) were included in the analysis. In all the included studies, PFA was performed using Farapulse system (Boston Scientific, Marlborough, MA) and the Farawave catheter, delivering 4 pairs of PFA applications for each pulmonary vein (PV) antrum in the “flower” and “basket” configurations. In half of the included studies, more PFA applications for PVI were delivered at operator's discretion [20, 22, 23]. In the RFA group, electroanatomical mapping was performed using CARTO system (Biosense Webster) in 4 studies [18, 19, 20, 21], Ensite Percision or Ensite X (Abbott) in the study by Wormann et al. [22] and both CARTO ed Ensite in the study by Popa et al. [23]. Four out six studies [19, 21, 22, 23] reported a vHPSD RFA strategy using two different ablation catheters, the QDOT Micro catheter (Biosense Webster) and the FlexAbility SE (Abbott), with RF delivery setting of 90 W/4 s and 70 W/7 or 5 s (anterior or posterior wall), respectively. The last 2 studies delivered RFA with the Thermocool SmartTouch SF catheter (Biosense Webster) with a power setting of 45 W in the study by Reinsch et al. [20] and 50 W in the study by Badertscher et al. [18]. Mean procedural time was 65.3 ± 38.9 min for PFA and 107.4 ± 39 min for HPSD/vHPSD, and mean fluoroscopy time was 15.3 ± 6.6 min for PFA and 6 ± 5.5 min for HPSD/vHPSD. Additional ablation lesions beyond PV were reported by Dello Russo et al. [19] in both the ablation strategy groups, whereas Soubh et al. [21] performed additional cavo‐tricuspid isthmus ablation in patients with concomitant atrial flutter. All procedural characteristics of the included studies are presented in Table 2. The quality of included studies was assessed using the NOS for non‐randomized cohort studies. All the studies presented a moderate‐to‐high quality as indicated by a NOS score ≥ 6 (Table 1).

**Table 1:** Baseline characteristics of the included studies (PFA vs HPSD/vHPSD ablation).

First author/year	Study design	Study population	n of patients (*n*) PFA vs HPSD/vHPSD	Age (years) PFA vs HPSD/vHPSD	Male (%) PFA vs HPSD/vHPSD	PAF (%) PFA vs HPSD/vHPSD	PersAF (%) PFA vs HPSD/vHPSD	AADs before ablation therapy PFA vs HPSD/vHPSD	LVEF (%) PFA vs HPSD/vHPSD	LA size PFA vs HPSD/vHPSD	AADs after CA	Primary outcome	Follow‐up (months)	Definition of recurrences	Blanking period (months)	AF recurrence monitoring	NOS
Popa/2023 [23]	Comparative, single center, non randomized retrospective observational study	Symptomatic PAF patients undergoing de novo CA (PFA, standard RFA*, vHPSD‐70W or vHPSD‐90W)	127 (35 vs 92)	61.6 ± 11.4 vs 63.5  10.6	68.6 vs 54.3	100 vs 100	0 vs 0	AADs were discontinued > 5 half‐lives before CA	58.5  2.7 vs 58.7 ± 5.9	NA	AADs were discontinued immediately after CA	Arrhythmia‐free survival during follow‐up	6	Any detected atrial arrhythmia (AF, AT)  30 s	2	12‐lead ECG, 5‐days Holter ECG	7
Wörmann/2023 [22]	Comparative, single center, non randomized retrospective observational study	Symptomatic PAF or persAF patients undergoing de novo CA (PFA or vHPSD)	114 (57 vs 57)	67 ± 13 vs 67 ± 12	33 vs 40	30 vs 30	70 vs 70	28% vs 23%	56 6 vs 56 ± 9	Diameter: 39.6 ± 6.0 mm vs 38.0  3.5 mm	AADs were discontinued immediately after PVI (PAF group)/after 3 months (persAF group)	Arrhythmia‐free survival during follow‐up	12	Any detected atrial arrhythmia (AF, AFL, AT) > 30 s	3	Resting ECG, 48‐h Holter ECG, photopletysmogram app‐based tele‐consultation, CIED interrogation	8
Dello Russo/2024 [19]	Comparative, multicentre, non randomized retrospective, observational study	PAF or persAF patients undergoing de novo or redo CA (PFA or vHPSD)	PSM: 342 (171 vs 171)	PSM: 65  10 vs 66 ± 12	PSM: 63 vs 65	PSM: 67 vs 68	PSM: 33 vs 32	NA	PSM: 60 ± 7 vs 60 ± 5	PSM: Indexed LA volume: 35  12 mL/m^2^ vs 34 ± 0 mL/m^2^	PSM: 27% vs 28%	Arrhythmia‐free survival during follow‐up	12	Any detected atrial arrhythmia (AT, AF, AFL) ≥ 30 s	1	12‐lead ECG, 24‐h Holter ECG	9
Reinsch/2024 [20]	Comparative, single center, non randomized retrospective, observational study	Symptomatic PAF patients undergoing de novo CA (PFA or HPSD)	410 (201 vs 209)	68 ± 14 vs 68± 16	56 vs 53	100 vs 100	0 vs 0	23% vs 21%	NA	NA	AADs were stopped 3 months after the initial ablation procedure if acceptable	Arrhythmia‐free survival during follow‐up	12	Any atrial tachyarrhythmia > 30 s identified on surface ECG or on Holter monitoring, off AADs	3	Clinical and 5‐day Holter ECG	8
Soubh/2024 [21]	Comparative, single center, non randomized retrospective, observational study	Symptomatic PAF or persAF patients undergoing CA (PFA or vHPSD)	82 (52 vs 30)	67 ± 10 vs 73 ± 7	71 vs 47	48 vs 43	52 vs 57	27 vs 17	52 ± 8 vs 47  14	Indexed LA volume: 39  14 mL/m^2^ vs 48 ± 18 mL/m^2^	NA	Arrhythmia‐free survival during follow‐up	6	NA	3	Clinical and 3‐day Holter ECG	6
Badertscher/2023 [18]	Comparative, single center, non randomized prospective, observational study	AF patients undergoing de novo CA (PFA or HPSD)	115 (52 vs 63)	65  10 (overall population)	NA	56% (overall population)	44% (overall population)	NA	56 ± 11 (overall population)	LA size: 41  6.6 mm (overall population)	NA	AF‐free survival during follow‐up	~7 (214 ± 273 days)	NA	NA	NA	6

*Note:* All values are displayed as mean ± standard deviation. Studies with six or more points (Newcastle‐Ottawa Scale) were regarded as having a high quality.

Abbreviations: AAD, antiarrhythmic drug; AF, atrial fibrillation; AFL, atrial flutter; AT, atrial tachycardia; CA, catheter ablation; CIED, cardiac implantable electronic device; ECG, electrocardiogram; HPSD, high‐power short‐duration; LA, left atrium; LVEF, left ventricular ejection fraction; NA, not available; NOS, Newcastle‐Ottawa Scale; PAF, paroxysmal atrial fibrillation; PersAF, persistent atrial fibrillation; PFA, pulsed field ablation; PSM, propensity score matching; PVI, pulmonary vein isolation; RFA, radiofrequency ablation; vHPSD, very high‐power short‐duration.

* Patients underwent standard RFA ablation were excluded from our analysis.

**Table 2:** Baseline procedural characteristics (PFA vs HPSD/vHPSD ablation).

First author/year	PFA catheter type	PFA additional characteristics	Additional ablation beyond PVI (PFA)	HPSD/vHPSD ablation	HPSD/vHPSD catheter type	HPSD/vHPSD additional characteristics	Additional ablation beyond PVI (HPSD/vHPSD)	Fluoroscopy duration (min)	Procedure duration (min)	LA dwell time (min)
Popa/2023 [23]	Farawave PFA Catheter	For each PV antrum, 4 PFA pulse cycles were applied in the “basket” configuration, followed by 4 pulse cycles in the “flower” configuration; more PFA applications were delivered at operator's discretion	no	vHPSD‐70 W/vHPSD‐90 W	4 mm irrigated‐tip catheter FlexAbility SE (vHPSD‐70W) or the 3.5 mm irrigated‐tip catheter QDOT Micro (vHPSD‐90W)	vHPSD‐70 W: 70 W/7 s (anterior wall) or 70 W/5 s (posterior wall), power‐ controlled mode. In case first‐pass PVI was not achieved, touch‐up lesions were applied. vHPSD‐90 W: 90 W/4 s (anterior and posterior wall) in a temperature‐controlled mode (QMODE + ). In case first‐pass PVI was not achieved, touch‐up RF lesions using 50 W/15 s were applied in the conventional QMODE.	no	PFA: 18.2  9.5 vs vHPSD‐70W: 7.0 ± 4.1 vs vHPSD‐90W: 6.0 ± 2.7	PFA: 81.1 ± 26.0 vs vHPSD‐70W: 106.7 ± 27.2 vs vHPSD‐90W: 112.8 ± 29.6	PFA: 59.1 ± 26.2 vs vHPSD‐70W: 85.7 ± 23.4 vs vHPSD‐90W: 84.4 ± 29.3
Wörmann/2023 [22]	Farawave PFA Catheter	At every PV antrum, 8 PFA impulses were delivered in the “flower” and “basket” configuration of the catheter; more PFA applications were delivered at operator's discretion	NA	vHPSD	A non contact‐force ablation catheter with enhanced tip irrigation (20 mL/min)	For circumferential PVI a power setting of 70 W for 7 s was used at all sites except for the posterior wall, were RF duration was reduced to 5 s	no	PFA: 15 ± 5 vs vHPSD: 12 3	PFA: 65 ± 17 vs vHPSD: 95 ± 23	NA
Dello Russo/2024 [19]	Farawave PFA Catheter	4 pairs of PFA applications in “basket “and “flower “configurations	The ablation of structures beyond PVs was performed in case of persAF with risk factors for recurrence (LA dilation or long duration of persAF episode) and in patients with LA low‐voltage areas or undergoing redo ablation	vHPSD	QDOT Micro catheter	In posterior segments of the PVs, vHPSD RF applications (QMODE + , 90 Watts/4 s, 8 mL/min flow rate; temperature controlled mode) were delivered. In anterior segments, operators either used pure vHPSD or temperature‐controlled 50 Watts pulses (QMODE) targeting AI values of 500‐550 (hybrid vHPSD).	The ablation of structures beyond PVs was performed in case of persAF with risk factors for recurrence (LA dilation or long duration of persAF episode) and in patients with LA low‐voltage areas or undergoing redo ablation	PSM: PFA: 15 ± 6 vs vHPSD: 7 ± 8	PSM: PFA: 70 ± 20 vs vHPSD: 100 ± 45	NA
Reinsch/2024 [20]	Farawave PFA Catheter	8 biphasic pulse trains with a power of 1900 or 2000 V were applied per PV (4 pairs of PFA applications in “basket “and “flower “configurations). More PFA applications were delivered at operator's discretion	no	HPSD	3.5 mm Thermocool Smarttouch SF catheter (Biosense Webster) with tip irrigation (15 mL/min during ablation)	Power control mode, 45 W. RF energy was delivered until an AI of 450 at the posterior wall/inferior/roof and 600 at the anterior wall was reached. In the case of absence of isolation after completion of the circle, touch‐up ablation was delivered until bidirectional PVI was achieved	no	PFA: 16 ± 7 vs HPSD: 4 3	PFA: 61 ± 59 vs HPSD: 125 38	PFA: 46 ± 58 vs HPSD: 110 ± 33
Soubh/2024 [21]	Farawave PFA Catheter	PFA energy was applied 8 times per PV at ostial positions, 4 times in “basket” and 4 times in “flower” configuration	All PFA procedures were combined with electroanatomical mapping. Additional CTI in patients with concomitant typical AFL	vHPSD	QDOT Micro catheter (Biosense Webster), Qmode+ (90 W/4 s)	Further ablation lines were applied if needed	Additional CTI in patients with concomitant typical AFL	PFA: 14 ± 6 vs vHPSD: 9 5	PFA: 64  19 vs vHPSD: 99 32	PFA: 41 ± 12 vs vHPSD: 62 ± 29
Badertscher/2023 [18]	Farawave PFA Catheter	PVI was performed with 4 applications in the “basket” configuration and 4 applications in the “flower” configuration per PV	no	HPSD	An irrigated‐tip ThermoCool catheter at 50 W (Smarttouch SF, Biosense Webster)	Single‐catheter ablation procedure	no	PFA: 13 ± 6 vs HPSD: 2.2 ± 2.3	PFA: 58 ± 18 vs HPSD: 83 ±28	NA

*Note:* All values are displayed as mean ± standard deviation.

Abbreviations: AFL, atrial flutter; AI, ablation index; CTI, cavotricuspid isthmus; HPSD, high‐power short‐duration; LA, left atrium; LAPW, left atrial posterior wall; NA, not available; PersAF, persistent atrial fibrillation; PFA, pulsed field ablation; PSM, propensity score matching; PV, pulmonary vein; PVI, pulmonary vein isolation; RF, radiofrequency; vHPSD, very high‐power short‐duration.

### 3.2 Clinical Outcomes

**Figure 1:**
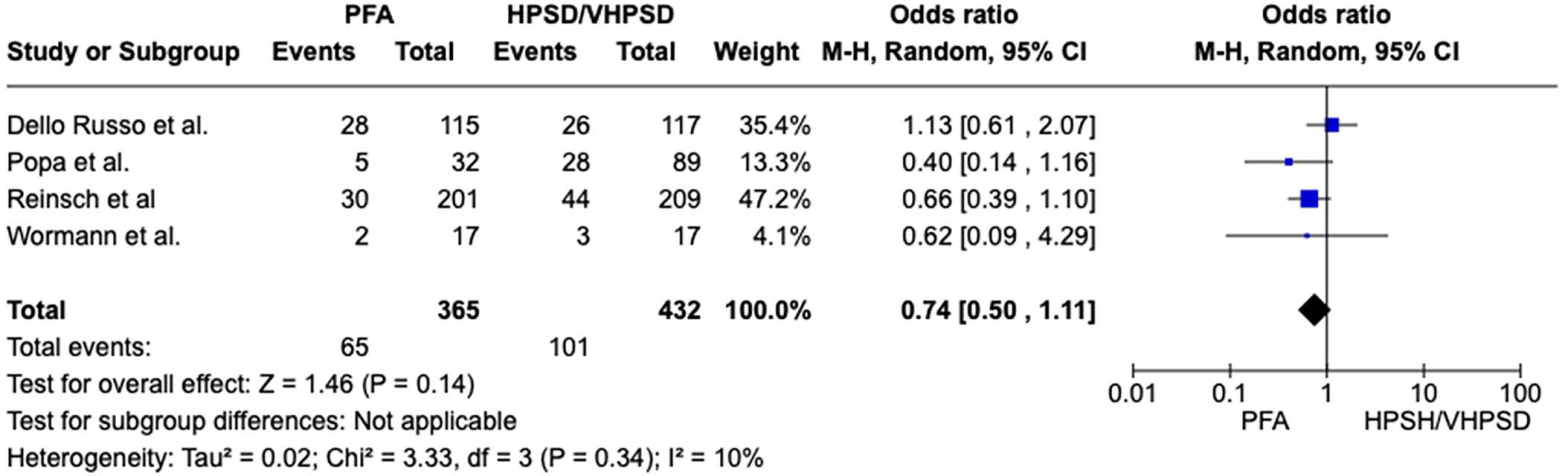
Comparison of AF recurrence between PFA and HPSD/vHPSD RFA in PAF patients. CI, confidence interval; HPSD, high‐power short‐duration; PFA, pulsed field ablation; vHPSD, very high‐power short‐duration.

**Figure 2:**
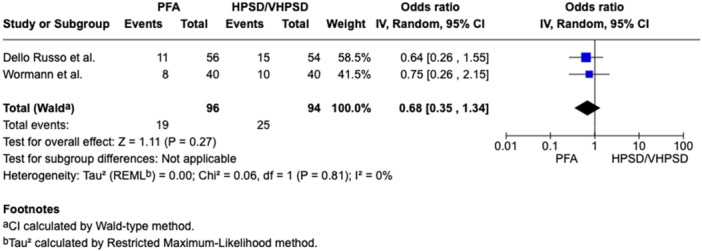
Comparison of AF recurrence between PFA and HPSD/vHPSD RFA in PeAF patients. CI, confidence interval; HPSD, high‐power short‐duration; PFA, pulsed field ablation; vHPSD, very high‐power short‐duration.

**Figure 3:**
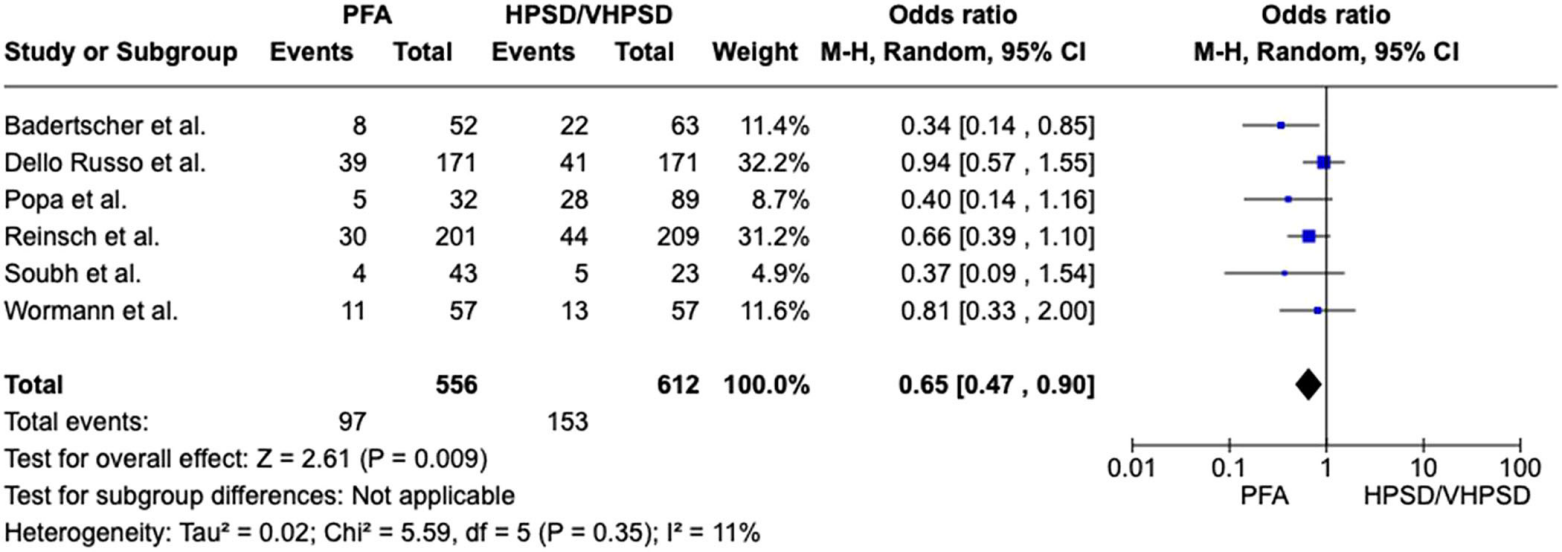
Comparison of AF recurrence between PFA and HPSD/vHPSD RFA in overall population. CI, confidence interval; HPSD, high‐power short‐duration; PFA, pulsed field ablation; vHPSD, very high‐power short‐duration.

**Figure 4:**
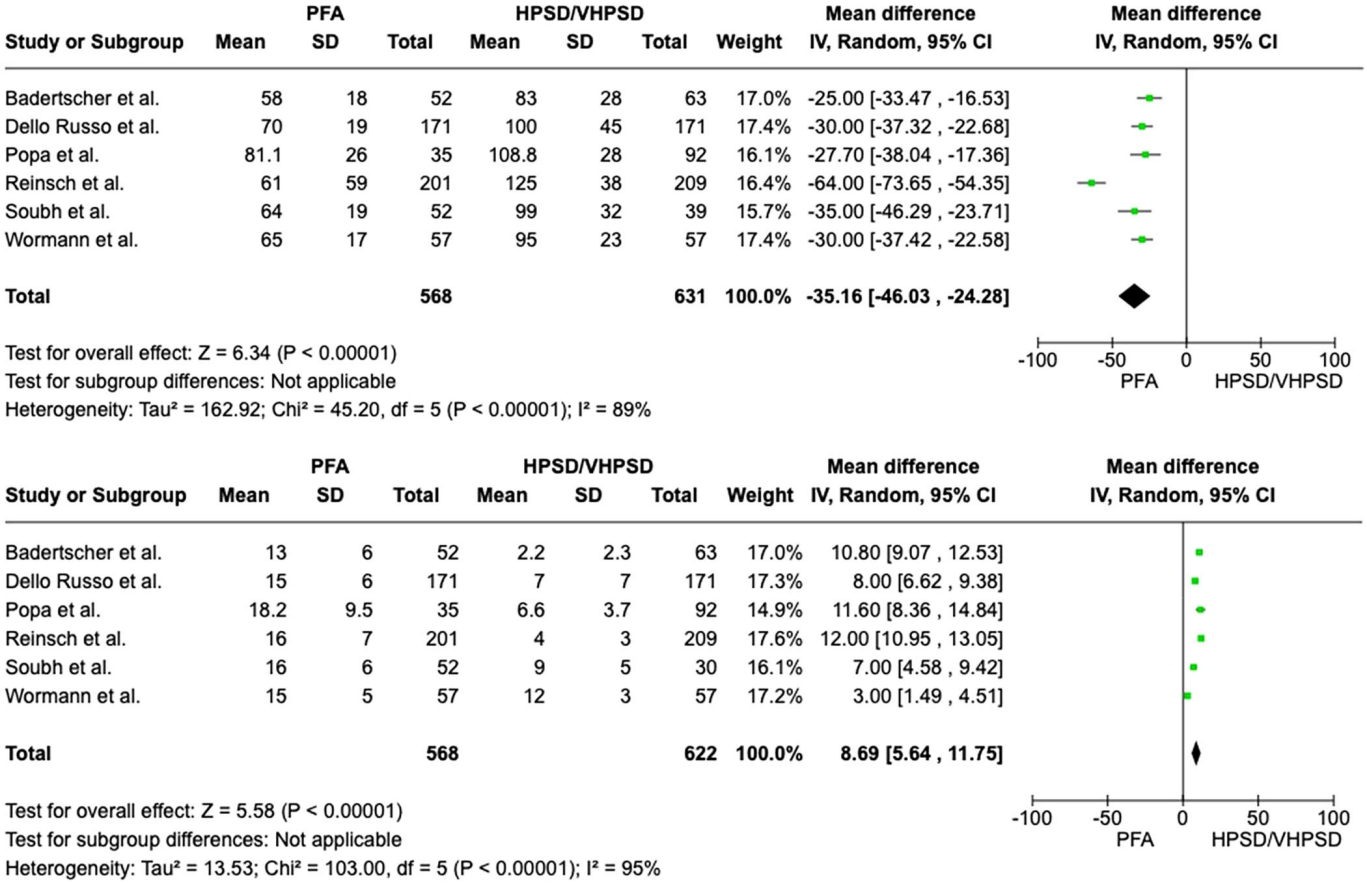
Comparison of procedure time (superior panel) and fluoroscopy time (inferior panel) between PFA and HPSD/vHPSD RFA. CI, confidence interval; HPSD, high‐power short‐duration; PFA, pulsed field ablation; vHPSD, very high‐power short‐duration.

**Figure 5:**
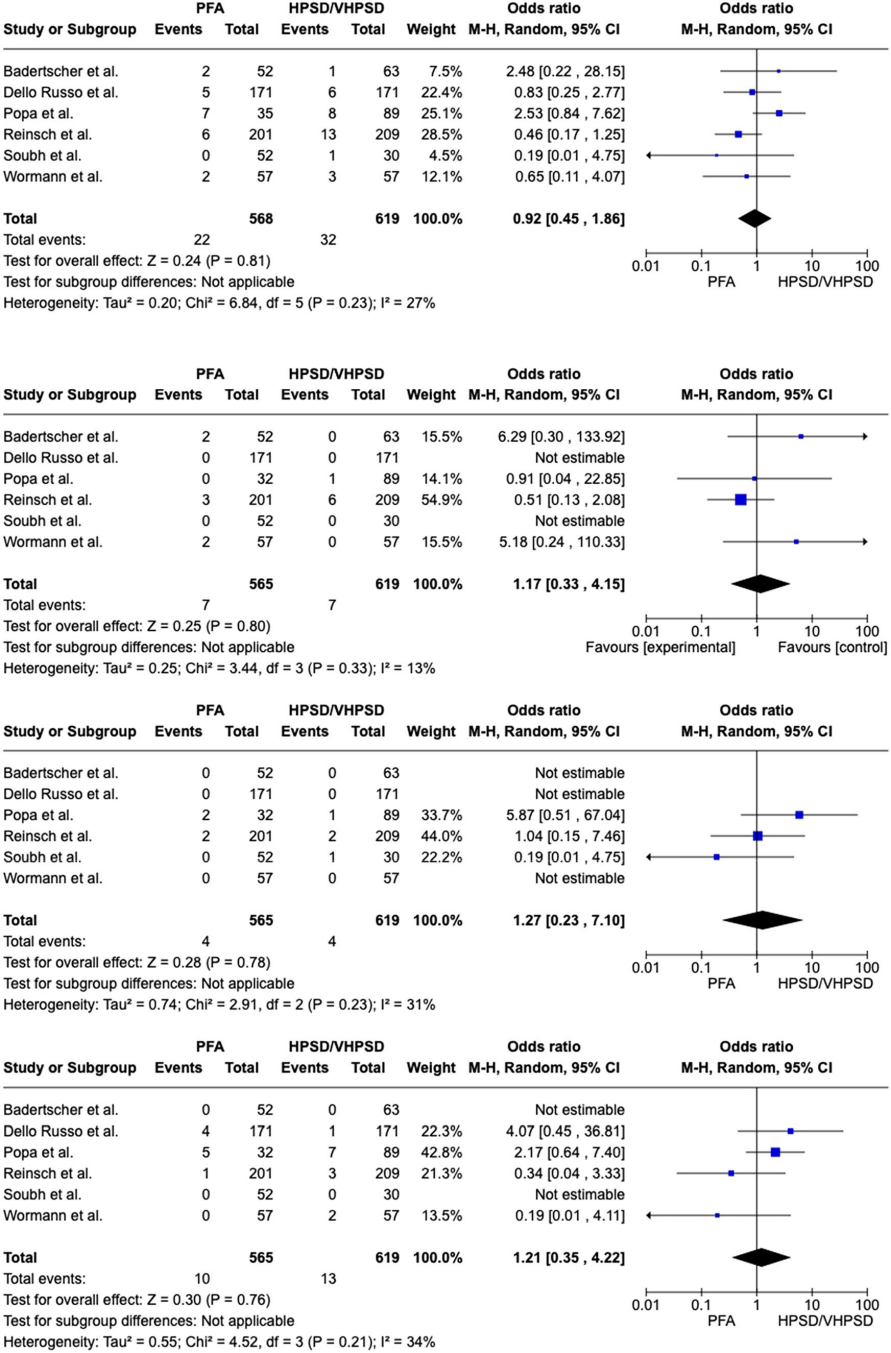
Comparison of overall complication, tamponade, stroke and vascular complications between PFA and HPSD/vHPSD RFA (from the top to the bottom). CI, confidence interval; HPSD, high‐power short‐duration; PFA, pulsed field ablation; vHPSD, very high‐power short‐duration.

In patients with PAF a nonsignificant 26% reduction of AF recurrence was related to PFA (recurrence rate 17.8%) as compared to HPSD/vHPSD RFA (recurrence rate 23.4%), with a pooled OR of 0.74 ([95%CI 0.50; 1.11], *p*‐value 0.14, I^2^ 10%) (Figure 1). In patients with PeAF a nonsignificant 32% reduction of AF recurrence was related to PFA as compared to HPSD/vHPSD RFA, with a pooled OR of 0.68 ([95%CI 0.35; 1.34], *p*‐value 0.27, I^2^ 10%) (Figure 2). In the overall population, PFA was associated with a significant reduction of AF recurrence at follow‐up as compared with HPSD/vHPSD RFA (OR 0.65 [95%CI 0.47; 0.90], *p*‐value 0.009, I^2^ 11%), with 17.4% AF recurrence rate after PFA and 25% recurrence rate after HPSD/vHPSD RFA (Figure 3). PFA was associated with shorter procedure time (WMD −35.16 [95% CI − 46.03; − 24.28], *p*‐value < 0.001, I^2^ = 89%) but longer fluoroscopy time (WMD 8.69 [95% CI 5.64; 11.75], *p*‐value < 0.001, I^2^ = 95%) (Figure 4) as compared with HPSD/vHPSD RFA. As for complications, there was no significant difference in overall complication rates between PFA (3.9%) and HPSD/vHPSD RFA (5.2%), with an OR 0.92, (95% CI 0.45;1.86, *p*‐value = 0.81, I^2^ = 27%) (Figure 5). No differences in terms of cardiac tamponade (*p*‐value 0.80), stroke (*p*‐value 0.78), and vascular complications (*p*‐value 0.76) were found between the two ablative strategies (Figure 4). As shown in Table 3, pericarditis were reported in the study by Dello Russo et al. (1 in PFA group vs 5 in vHPSD RFA group, *p*‐value 0.215) [19], whereas transient ST‐segment elevation were reported by Reinsch et al. [20] and Badertscher et al. [18] only in HPSD group (OR 0.28 [95% CI 0.03;2.56], *p*‐value 0.26, I^2^ 0%). The sensitivity analysis using a fixed effect model showed comparable results as compared with the random effect model analysis in terms of AF recurrence rates in the overall population and in the PAF population, as well as in terms of procedural and fluoroscopy time and complication rates (Supporting Information Figures A.2–A.9). In overall population, the significant reduction of AF recurrence associated with PFA as compared with HPSD/vHPSD RFA was maintained after excluding studies with additional ablation beyond PVI (OR 0.57 [95%CI 0.39; 0.83], *p*‐value 0.004, I^2^ 0%) (Supporting Information Figures A.10*–*A.11). PFA use was associated with significant reduction of AF recurrences at the leave‐one‐out sensitivity analyses after excluding any of the studies on turn, Badertscher et al. (*p*‐value 0.004), Dello Russo et al. (*p*‐value 0.002), Popa et al. (*p*‐value 0.03), Reinsch et al. (*p*‐value 0.04), Soubh et al. (*p*‐value 0.02) and Wormann et al. (*p*‐value 0.01) (Supporting information Figures A.12–A.17). The sub‐analysis including studies with 6–7 months of follow‐up [18, 21, 23] showed a significant reduction of AF recurrence associated with PFA as compared with HPSD/vHPSD (OR 0.37 [95% CI 0.20; 0.68], *p*‐value 0.002, I^2^ 0%), whereas a nonsignificant 21% reduction of AF recurrence was found when including studies with longer follow‐up duration (OR 0.79 [95% CI 0.57; 1.10], *p*‐value 0.17, I^2^ 0%) (Supporting Information Figures A.18–A.19). In Figure A.20 we reported the efficacy outcome in PAF patients withdrawing AAD therapy after ablation procedure.

**Table 3:** Procedural complications (PFA vs HPSD/vHPSD ablation).

First author/year	Tamponade (*n*)	Stroke/TIA (*n*)	Air embolism (*n*)	PV stenosis (*n*)	Death (*n*)	Vascular complications (*n*)	Atrioesophageal fistula (*n*)	Pericarditis (*n*)	Transient ST segment elevation (*n*)
Popa/2023 [23]	PFA: 0 vHPSD‐70W: 0 vHPSD‐90W: 0	PFA: 2 vHPSD‐70W: 0 vHPSD‐90W: 1	NA	NA	PFA: 0 vHPSD‐70W: 0 vHPSD‐90W: 0	PFA: 5 vHPSD‐70W: 3 vHPSD‐90W: 4	PFA: 0 vHPSD‐70W: 0 vHPSD‐90W: 0	NA	NA
Wörmann/2023 [22]	PFA: 2 vHPSD: 0	NA	NA	PFA: 0 vHPSD: 1	PFA: 0 vHPSD: 0	PFA: 0 vHPSD: 2	NA	NA	NA
Dello Russo/2024 [19]	PSM: PFA: 0 vHPSD: 0	PSM: PFA: 0 vHPSD: 0	NA	PSM: PFA: 0 vHPSD: 0	PSM: PFA: 0 vHPSD: 0	PFA: 4 vHPSD: 1	PSM: PFA: 0 vHPSD: 0	PSM: PFA: 1 vHPSD: 5	NA
Reinsch/2024 [20]	PFA: 3 HPSD: 6	PFA: 2 HPSD: 2	PFA: 0 HPSD: 2	PFA: 0 HPSD: 0	PFA: 1 HPSD: 0	PFA: 1 HPSD: 3	PFA: 0 HPSD: 0	NA	PFA: 0 HPSD: 2
Soubh/2024 [21]	PFA: 0 vHPSD: 0	PFA: 0 vHPSD: 1	NA	NA	PFA: 0 vHPSD: 0	PFA: 0 vHPSD: 0	PFA: 0 vHPSD: 0	NA	
Badertscher/2023 [18]	PFA: 2 HPSD: 0	PFA: 0 HPSD: 0	PFA: 0 HPSD: 0	PFA: 0 HPSD: 0	PFA: 0 HPSD: 0	PFA: 0 HPSD: 0	PFA: 0 HPSD: 0	PFA: 0 HPSD: 0	PFA: 0 HPSD: 1

Abbreviations: HPSD, high‐power short‐duration; NA, not available; PFA, pulsed field ablation; PSM, propensity score matching; PVI, pulmonary vein; TIA, transient ischemic attack; vHPSD, very high‐power short‐duration.

## 4 Discussion

Herein, we present a meta‐analysis of studies comparing the efficacy and safety of PFA vs HPSD/vHPSD RFA in patients undergoing AF ablation. The main findings of the current meta‐analysis can be resumed as follows:

- In PAF patients, PFA led to a nonsignificant 26% reduction of AF recurrence as compared with HPSD/vHPSD RFA;
- In patients with PeAF a nonsignificant 32% reduction of AF recurrence was related to PFA as compared to HPSD/vHPSD RFA, whereas in the overall population PFA was associated with a significant reduction of AF recurrence at follow‐up as compared with HPSD/vHPSD RFA, with 17.4% AF recurrence rate after PFA and 25% recurrence rate after HPSD/vHPSD RFA;
- PFA was associated with a significant increase in terms of fluoroscopic time and a significant decrease in procedural time as related to HPSD/vHPSD RFA;
- No differences in terms of peri‐procedural complications were observed among the 2 strategies.

Nowadays, catheter ablation targeting PVI is considered the most effective rhythm control strategy for AF. The suboptimal long‐term results achieved with conventional point‐by‐point AF RFA fueled technological research and advancements leading to the development of innovative forms of ablation such as HPSD/vHPSD RFA and PFA.

On the one hand, HPSD/vHPSD RFA with > 40 W power and shorter RF application time has been proposed as alternative to conservative power mode to increase safety and reduce duration of AF ablation, with some beneficial differences in terms of lesion biophysics [26]. Indeed, the shorter RF delivery of HPSD/vHPSD increases resistive heating of the surface tissue layer and reduces the time‐dependent conductive heating, resulting in shallower but wider lesions, thus improving lesion‐to‐lesion uniformity and linear contiguity [12, 26, 27, 28, 29, 30]. However, clinical trials comparing HPSD and conventional RFA strategies yielded conflicting results, without demonstrating clear superiority of the newer technique in terms of safety and efficacy in AF ablation [12, 26, 29, 30].

On the other hand, PFA is non‐thermal energy source that leads to cell death via electroporation. The delivery of ultrarapid electrical pulses results in the formation of membrane pores affecting electrical conductivity and membrane permeability, eventually resulting in cell death [31]. The negligible thermal effect of PFA and the different thresholds of vulnerability of noncardiac structures to PFA result in less collateral damage to structures as esophagus and phrenic nerve [31, 32]. In a recent meta‐analysis of 9 studies comparing PFA vs thermal energy ablation, Iqbal et al. [33] did not find differences in terms of AF recurrences at follow‐up as compared to thermal energy ablation. Conversely, our meta‐analysis showed a significant reduction of AF recurrences in overall population with PFA as compared with HPSD/vHPSD (OR 0.65 [95%CI 0.47; 0.90], *p*‐value 0.009, I^2^ 11%), although no differences were found when analyzing PAF and PeAF patients separately. The finding of improved efficacy of PFA in AF ablation compared to HPSD/vHPSD is hypothesis‐generating. Although Kawamura et al. [34] did not find differences in terms of PV antral isolation areas among PFA and thermal ablation, recent studies on lesion transmurality on left atrial posterior wall (LA‐PW) associated with PFA and HPSD/vHPSD may support our findings of improved efficacy with PFA showing a trend towards greater transmurality with PFA compared with HPSD/vHPSD RFA [35, 36, 37, 38]. Since non‐transmural lesion are associated with arrhythmia recurrence creating a substrate for endo‐epicardial dissociation and transmural re‐entry [37], the reduction of AF recurrence achieved by PFA as compared with HPSD/vHPSD RFA found in our meta‐analysis may be related to the lack of transmurality obtained by HPSD/vHPSD.

From a safety standpoint, we did not find differences in complication rate among PFA (3.9%) and HPSD/vHPSD RFA (5%), with an OR 0.92 (95% CI 0.45; 1.86, *p*‐value = 0.81, I^2^ = 27%), whereas the previous meta‐analysis by Aldaas et al. [17] found significantly fewer periprocedural complications in the PFA as compared with thermal ablation strategies. In this regard, the recently published MANIFEST‐17k study did not report esophageal damage, permanent phrenic palsy and PV stenosis among 17 642 patients undergone PFA for AF, underscoring the favorable safety profile of the technique [32]. de Campos et al. [16] found lower esophageal injury rates and higher tamponade rates after PFA as compared with thermal ablation strategies. This meta‐analysis included studies published in the early phase of PFA diffusion, with predominant use of standard RF and cryoballoon as thermal energy strategies [16]. Conversely, we did not find differences in tamponade rates among PFA (1.2%) and HPSD/vHPSD RFA (1.1%). The similar rates of cardiac tamponade among groups in our analysis, including more recently published studies, may be related to the universally transition to a J‐tip guidewire in substitution of the extra stiff guidewire used in the early phases of PFA treatment and considered responsible for the more frequent cases of cardiac tamponade found earlier. In line with the meta‐analysis by de Campos et al. [16] we did not find esophageal injuries in PFA group. However, de Campos et al. [16] reported 1.6% of esophageal injuries in thermal ablation group. In contrast, no esophageal injuries were reported in HPSD/vHPSD RFA group of our analysis, as expected by the different lesion biophysics associated with HPSD/vHPSD RFA settings resulting in shallower lesions [28]. However, the study is unpowered to draw solid conclusions on esophageal safety due to the limited sample size of the meta‐analysis and the lack of esophageal outcomes in both study groups. Moreover, the risk of severe esophageal injuries with RFA may be reduced using active esophageal cooling technologies, such as Attune Medical Esophageal Heat Transfer Device (EnsoETM). In a small RCT, Tschabrunn et al. [39] reported reduced rates of severe endoscopically detected esophageal thermal lesions in AF patients undergoing first‐time RFA with esophageal cooling device as compared with control group. Leung et al. [40] found significantly lower rates of esophageal mucosal injury in patients using a esophageal cooling device related to the standard sensor temperature probe. No differences in terms of vascular complications and stroke were observed among the groups, although the POWER‐FAST III trial recently reported an increased risk of embolic events with vHPSD as compared with conventional RF [29] and the SHORT‐AF trial revealed a trend towards increased rate of asymptomatic cerebral emboli (ACE) with HPSD RFA [12]. Since none of the studies included in the current meta‐analysis evaluated ACE by brain magnetic resonance, no definitive conclusions can be drawn by our results in terms of cerebrovascular safety of HPSD/vHPSD RFA. However, stroke rates were low and comparable among the study groups in the current analysis, similarly to the results of previous meta‐analysis comparing PFA vs standard RFA and cryoballoon ablation [16]. Table 3 presents the procedural complications observed in the analyzed studies.

In line with previous reports, PFA was associated with shorter procedure time and longer fluoroscopy time [16, 17], due to the single‐shot design of the PFA catheter and lack of integration with electroanatomic mapping systems, respectively. The longer fluoroscopy time is of concern because of the risks for patients and operators associated with longer exposure to ionizing radiation. However, the fluoroscopy exposure issue is expected to be solved with the soon available integration of electroanatomical mapping systems into PFA.

When comparing healthcare system efficiency and cost efficiency, PFA using a pentaspline multipolar catheter was associated with higher overall median costs as compared with RFA due to substantially higher equipment costs [41]. PFA burden on healthcare resources should be kept in mind when choosing among technologies with similar efficacy and safety.

Overall, the current meta‐analysis shows that PFA is associated with trend towards lower rates of arrhythmia recurrences at follow‐up as compared with HPSD/vHPSD RFA, without increasing complication risk. However, our results must be interpreted with caution. Indeed, the relatively small number of included patients and the short follow‐up entail confirmation of our results in further larger, well‐designed RCTs.

## 5 Limitations

Our meta‐analysis has several limitations. To begin with, we included 6 non‐randomized studies and some of them with small sample size, characterized by the use of different HPSD/vHPSD RFA settings and mapping techniques. However, RCTs comparing PFA to HPSD/vHPSD RFA are currently lacking, and we included the best evidence available on the topic. Secondly, the included studies adopted different blanking periods, AAD therapy durations, and follow‐up period. However, no statistical heterogeneity was found when analyzing the outcome AF recurrence. In addition, follow‐up duration of the included studies was up to 12 months, thus our results need to be confirmed by longer‐term follow‐up studies. Moreover, AF recurrence rates may have been underestimated because asymptomatic episodes may be undetected when using ECG and Holter monitoring. However, this should underestimate recurrences in both treatment arms. Eventually, all the included studies used FARAWAVE catheter for PFA, limiting the generalizability of our results to other types of PFA catheters.

## Conclusions

PFA in AF patients is associated with similar efficacy and safety profiles as compared to HPSD/vHPSD RFA, with shorter procedural but longer fluoroscopy times. Large, well‐designed RCTs are eagerly awaited to expand our findings.

## Conflicts of Interest

The authors declare no conflicts of interest.

## Supporting information

Supporting Material META ENERGY Reviewed.

## Data Availability

Data sharing is not applicable to this article as no new data were created or analyzed in this study.
