# Supplementary material for: Pentaspline Pulsed Field Ablation Versus High‐Power Short‐Duration/Very High‐Power Short‐Duration Radiofrequency Ablation in Atrial Fibrillation: A Meta‐Analysis
Source: J Cardiovasc Electrophysiol. 2025 Jul 9;36(9):2165–78. doi: 10.1111/jce.16776 (PMC12420869; doi:10.1111/jce.16776)
Supplement: Supplementary file 1 — Supporting Material META ENERGY Reviewed. [file JCE-36-2165-s001.docx]

1009 records identified by searching in online libraries

659 records excluded after duplicated or unrelated data

Records screened

(n = 350 )

Records excluded based on title and/or abstract

(n = 337)

Reports excluded after applying the inclusion and exclusion criteria (n = 7):

- No endpoints of interest (n = 2)
- No full text (n = 4)
- Duplicated data (n = 1)

Full-text articles assessed for eligibility

(n = 13)

6 studies included in the quantitative syntesis and meta-analysis

**Figure A.1.** Study flow diagram.

**
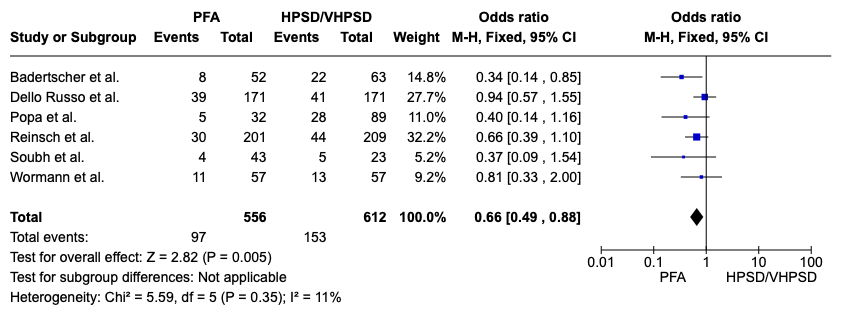
**

**Figure A.2.** Forest plot of atrial fibrillation recurrence in overall population (fixed effect model). CI: confidence interval; HP-SD/vHP-SD: high-power short-duration/ very high-power short-duration; PFA: pulsed field ablation.


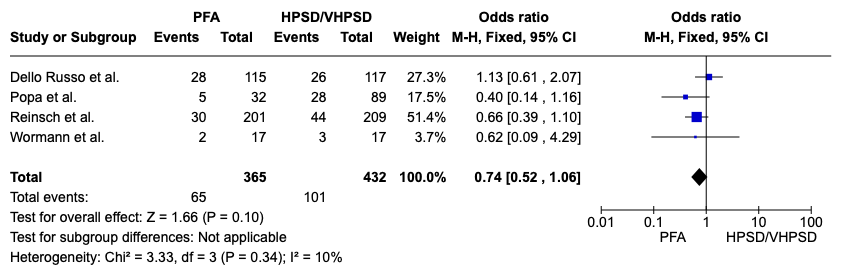


**Figure A.3.** Forest plot of atrial fibrillation recurrence in paroxysmal atrial fibrillation patients (fixed effect model). CI: confidence interval; HP-SD/vHP-SD: high-power short-duration/ very high-power short-duration; PFA: pulsed field ablation.


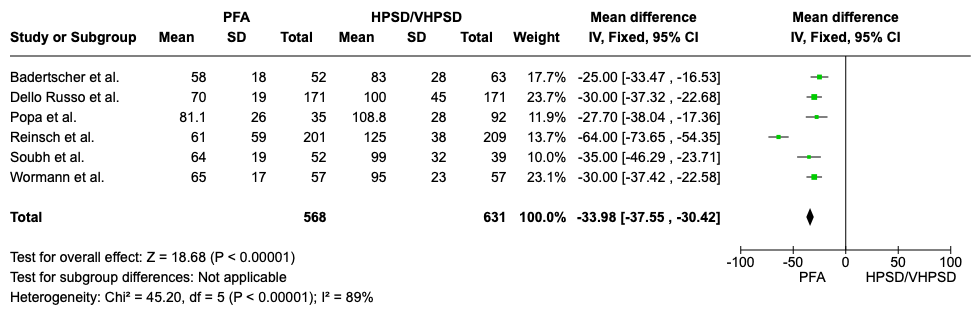


**Figure A.4.** Forest plot of procedural time (fixed effect model). CI: confidence interval; HP-SD/vHP-SD: high-power short-duration/ very high-power short-duration; PFA: pulsed field ablation.


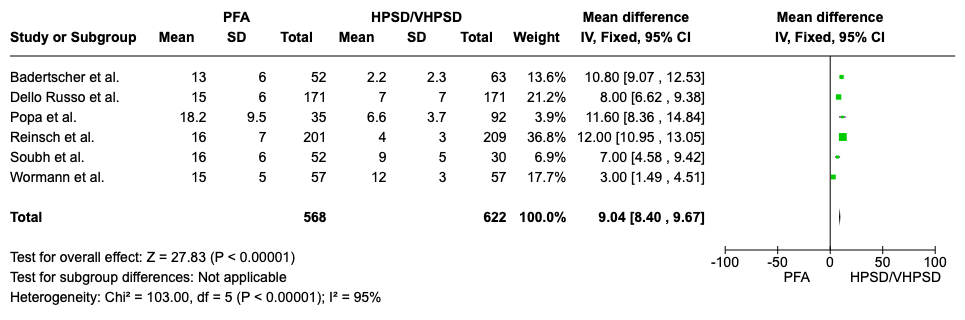


**Figure A.5.** Forest plot of fluoroscopy time (fixed effect model). CI: confidence interval; HP-SD/vHP-SD: high-power short-duration/ very high-power short-duration; PFA: pulsed field ablation.


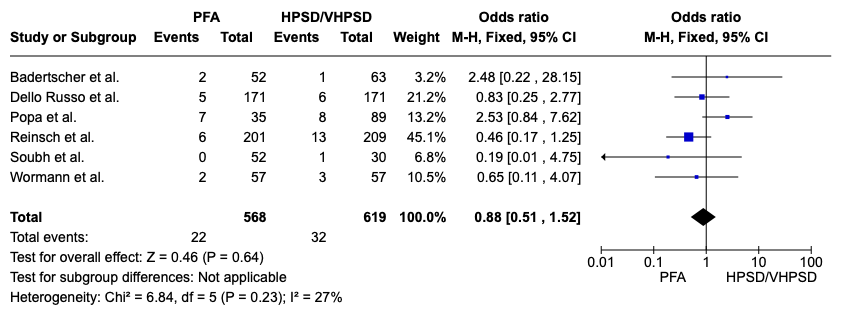


**Figure A.6.** Forest plot of overall complications (fixed effect model). CI: confidence interval; HP-SD/vHP-SD: high-power short-duration/ very high-power short-duration; PFA: pulsed field ablation.


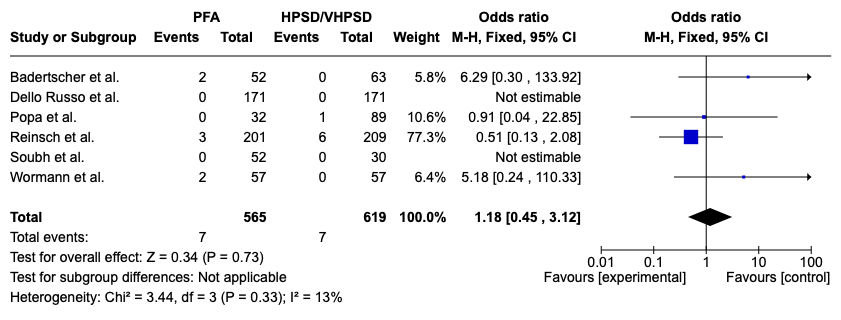


**Figure A.7.** Forest plot of cardiac tamponade (fixed effect model). CI: confidence interval; HP-SD/vHP-SD: high-power short-duration/ very high-power short-duration; PFA: pulsed field ablation.


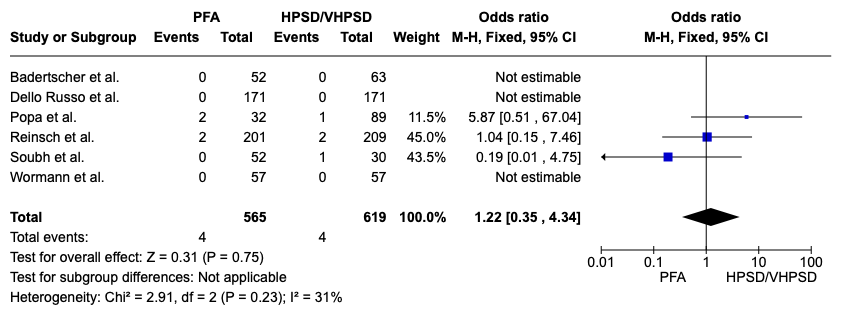


**Figure A.8.** Forest plot of stroke (fixed effect model). CI: confidence interval; HP-SD/vHP-SD: high-power short-duration/ very high-power short-duration; PFA: pulsed field ablation.


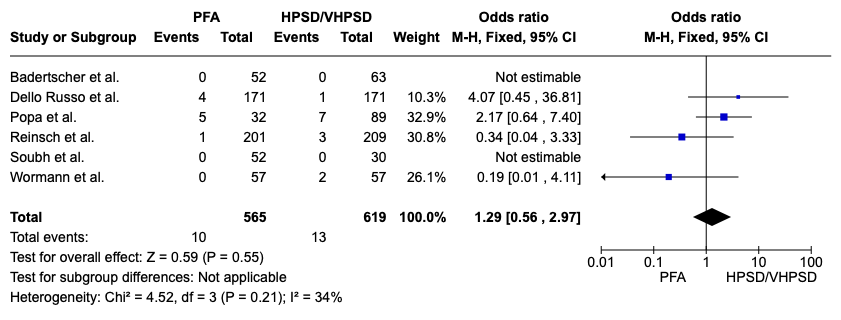


**Figure A.9.** Forest plot of vascular complications (fixed effect model). CI: confidence interval; HP-SD/vHP-SD: high-power short-duration/ very high-power short-duration; PFA: pulsed field ablation.


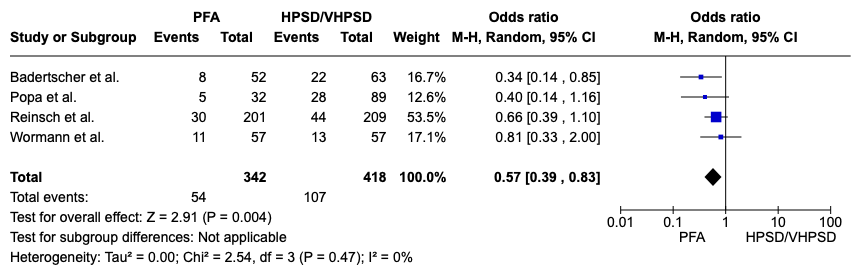


**Figure A.10.** Forest plot of atrial fibrillation recurrence in studies only performing PVI (random effect model). CI: confidence interval; HP-SD/vHP-SD: high-power short-duration/ very high-power short-duration; PFA: pulsed field ablation; PVI: pulmonary vein isolation.


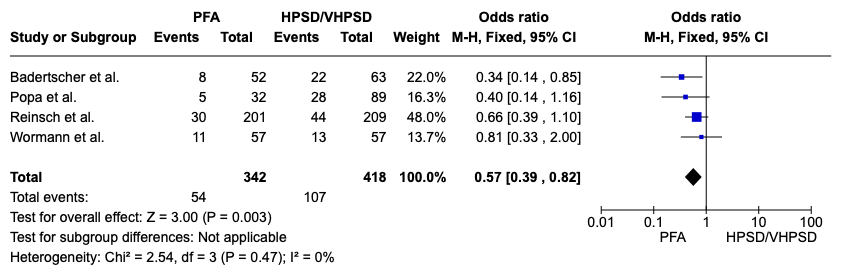


**Figure A.11.** Forest plot of atrial fibrillation recurrence in studies only performing PVI (fixed effect model). CI: confidence interval; HP-SD/vHP-SD: high-power short-duration/ very high-power short-duration; PFA: pulsed field ablation; PVI: pulmonary vein isolation.


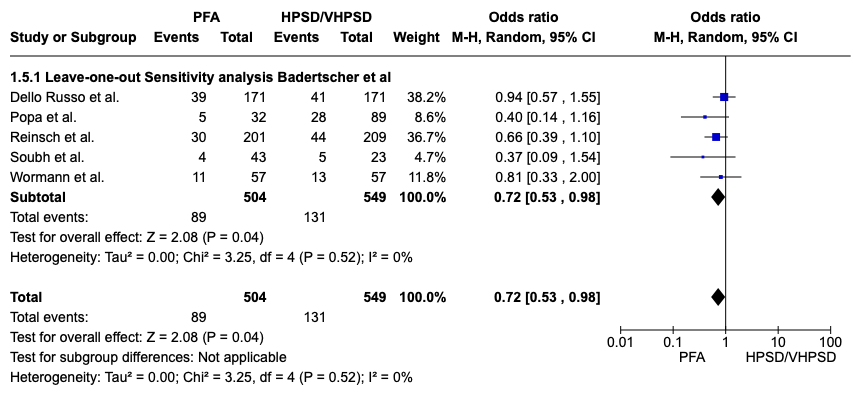


**Figure A.12.** Forest plot of atrial fibrillation recurrence excluding the study by Badertscher et al. (random effect model). CI: confidence interval; HP-SD/vHP-SD: high-power short-duration/ very high-power short-duration; PFA: pulsed field ablation.


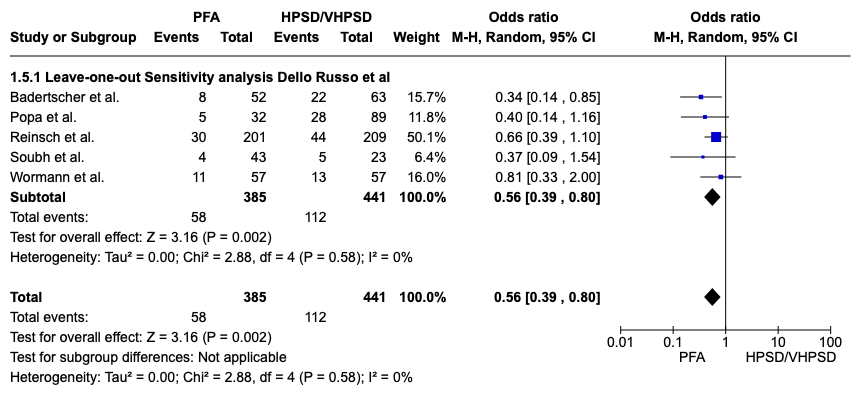


**Figure A.13.** Forest plot of atrial fibrillation recurrence excluding the study by Dello Russo et al. (random effect model). CI: confidence interval; HP-SD/vHP-SD: high-power short-duration/ very high-power short-duration; PFA: pulsed field ablation.


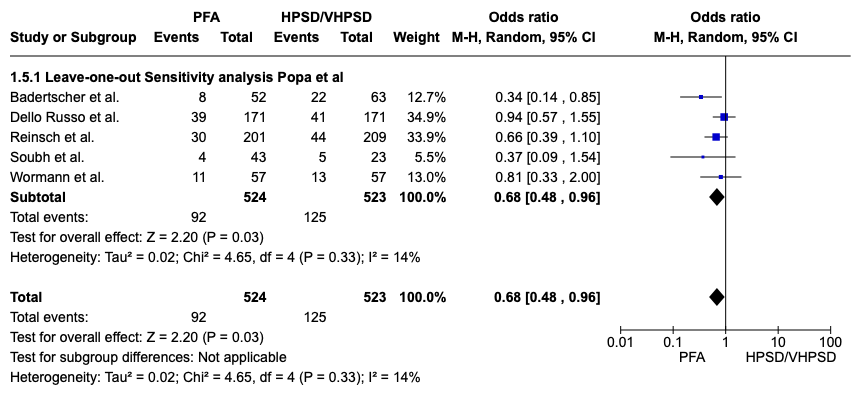


**Figure A.14.** Forest plot of atrial fibrillation recurrence excluding the study by Popa et al. (random effect model). CI: confidence interval; HP-SD/vHP-SD: high-power short-duration/ very high-power short-duration; PFA: pulsed field ablation.

**
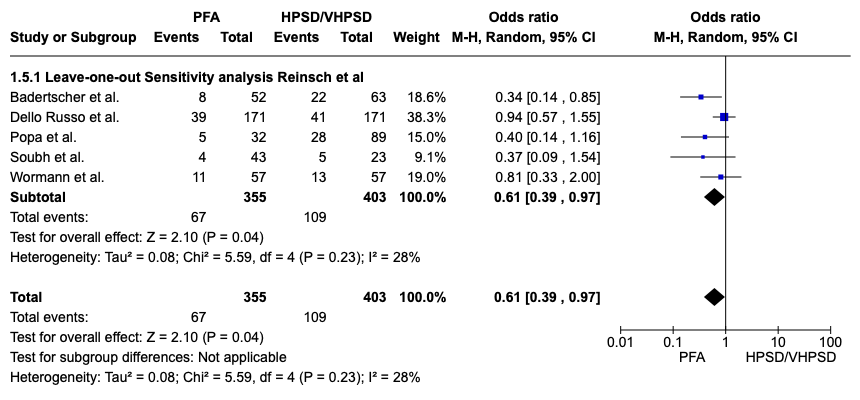
**

**Figure A.15.** Forest plot of atrial fibrillation recurrence excluding the study by Reinsch et al. (random effect model). CI: confidence interval; HP-SD/vHP-SD: high-power short-duration/ very high-power short-duration; PFA: pulsed field ablation.


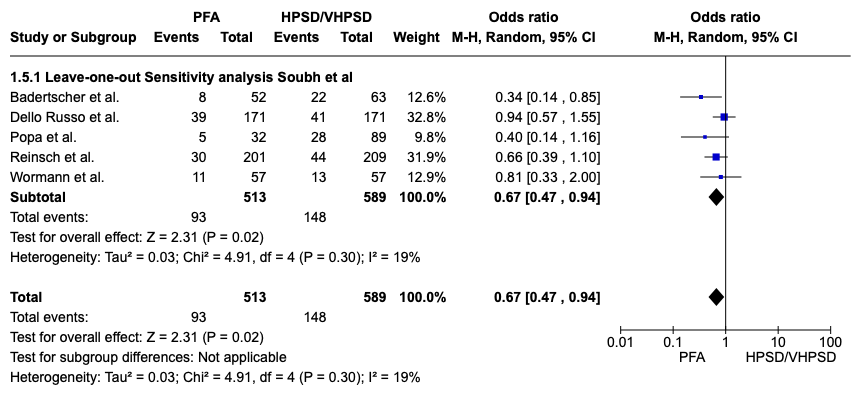


**Figure A.16.** Forest plot of atrial fibrillation recurrence excluding the study by Soubh et al. (random effect model). CI: confidence interval; HP-SD/vHP-SD: high-power short-duration/ very high-power short-duration; PFA: pulsed field ablation.


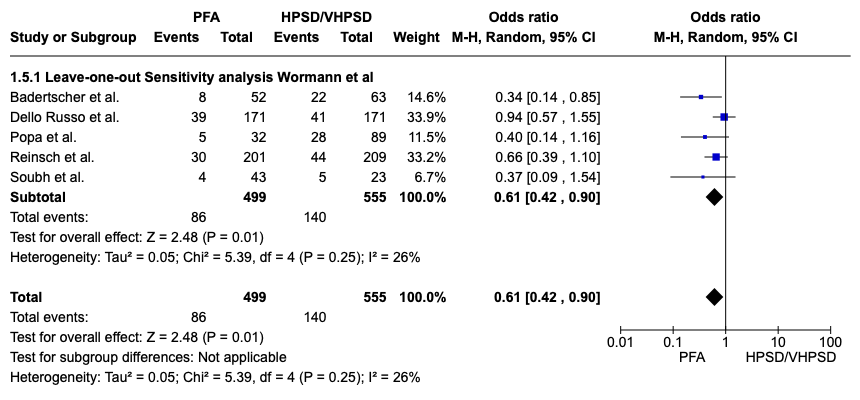


**Figure A.17.** Forest plot of atrial fibrillation recurrence excluding the study by Wormann et al. (random effect model). CI: confidence interval; HP-SD/vHP-SD: high-power short-duration/ very high-power short-duration; PFA: pulsed field ablation.


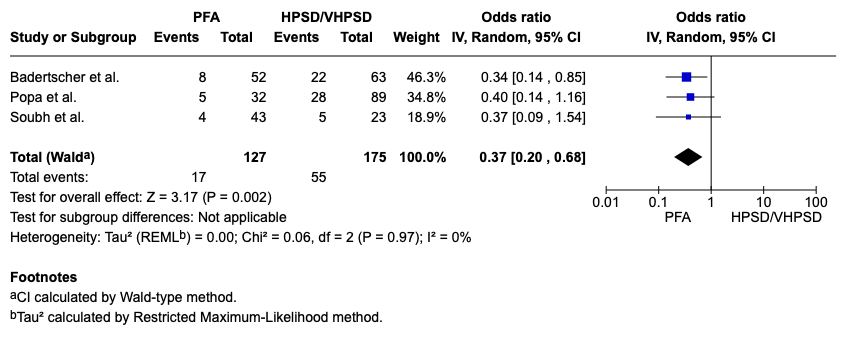


**Figure A.18**. Forest plot of atrial fibrillation recurrence including studies with short follow-up duration (random effect model). CI: confidence interval; HP-SD/vHP-SD: high-power short-duration/ very high-power short-duration; PFA: pulsed field ablation.


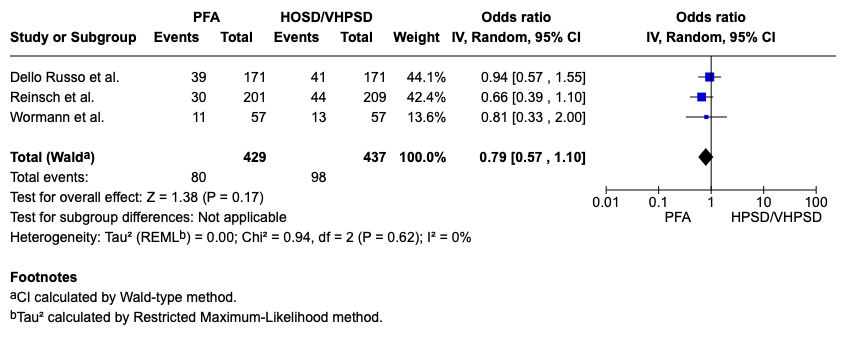


**Figure A.19**. Forest plot of atrial fibrillation recurrence including studies with 12 months follow-up (random effect model). CI: confidence interval; HP-SD/vHP-SD: high-power short-duration/ very high-power short-duration; PFA: pulsed field ablation.


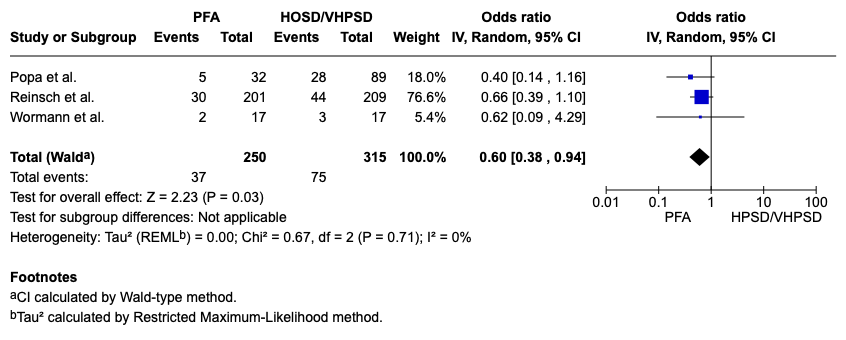


**Figure A.20**. Forest plot of atrial fibrillation recurrence including PAF patients withdrawing AADs after the procedure (random effect model). AADs: antiarrhythmic drugs; CI: confidence interval; HP-SD/vHP-SD: high-power short-duration/ very high-power short-duration; PFA: pulsed field ablation.
